# Aortic repair following initial decompressive craniectomy for acute type A aortic dissection complicated with extensive hemorrhagic cerebral infarction: a case report

**DOI:** 10.1186/s40792-022-01526-2

**Published:** 2022-09-19

**Authors:** Masaki Kano, Akinari Iwahori, Hitoshi Ogino

**Affiliations:** Department of Cardiovascular Surgery, Tokyo Medical University, 6-7-1 Nishishinjuku Shinjuku-ku, Tokyo, 160-0023 Japan

**Keywords:** Acute aortic dissection, Cerebral malperfusion, Cerebral infarction, Decompressive craniectomy, Hemiarch replacement

## Abstract

A 69-year-old woman presented with acute type A aortic dissection complicated by extensive hemorrhagic cerebral infarction due to brain malperfusion. Emergency decompressive craniectomy was initially performed, with an initial diagnosis of hemorrhagic cerebral infarction. The patient was referred for surgical management following a diagnosis of acute type A aortic dissection. After stabilizing the neurological condition with medical treatment for nine weeks, hemiarch replacement was performed electively. The postoperative course was uneventful, with no new neurological disorders. Subsequently, she recovered sufficiently to have daily conversations and attend hospital appointments using a wheelchair.

## Introduction

Acute type A aortic dissection (AAAD) is a lethal condition, which often requires emergency surgery. In addition, it is occasionally complicated with cerebral malperfusion; 4.6% of patients present with cerebrovascular accidents and 2.9% with coma [1]. In these cases, emergency aortic repair is controversial because of the high rates of severe cerebral morbidities and mortalities. Here, we report a rare case of aortic repair following decompressive craniectomy for AAAD after primary decompression craniectomy for extensive hemorrhagic cerebral infarction.

## Case report

**Fig. 1:**
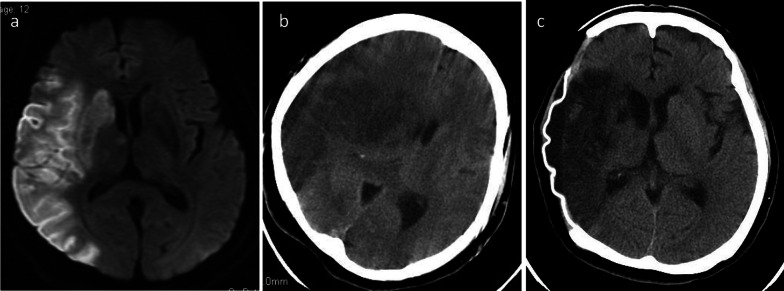
Brain MRI shows massive cerebral infarction of the right middle cerebral artery area (**a**). Brain CT shows diffuse brain edema resulting in brain herniation (**b**), and no cerebral edema after external decompression (**c**)

**Fig. 2:**
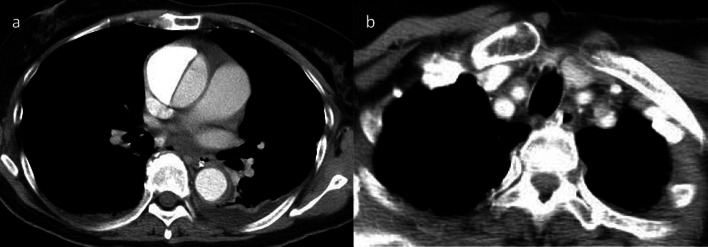
Chest CT shows double-barrel aortic dissection on the day of onset (**a**) and the dissected right common carotid artery with the false lumen thrombosed (**b**)

A 69-year-old woman suddenly lost consciousness and presented with left hemiplegia. After emergency transfer to a nearby hospital, head magnetic resonance imaging (MRI) revealed acute cerebral infarction in the perfusion area of the right middle cerebral artery (Fig. 1a). Her consciousness worsened despite conservative treatment for cerebral infarction. Brain computed tomography (CT) showed extensive hemorrhagic cerebral infarction and cerebral herniation accompanied by severe brain edema (Fig. 1b). An emergency decompressive craniectomy with an artificial dura was performed (Fig. 1c). On the 6th hospital day, radiologic interpretations of the body CT at admission were reported, and AAAD was first recognized. The final diagnosis of extensive cerebral infarction due to severe cerebral malperfusion caused by AAAD was established. Her general conditions, including neurological findings, became stable after the primary decompressive craniectomy. She was referred for surgical management of AAAD 9 days after the AAAD onset and was transferred to our university hospital after a further 2 days. At admission, she had great difficulty in communication (Glasgow Coma Scale [GCS] E4V1M1) and her left arm and leg were completely paralyzed (Manual Muscle Testing: MMT 1). Her systolic blood pressure was well controlled at 120 mmHg or less with continuous intravenous injection of a calcium blocker. Enhanced CT revealed classical aortic dissection consisting of a dilated, double-barreled aorta with the entry of the proximal aortic arch and extended dissection in the brachiocephalic and right common carotid arteries (Fig. 2). Preoperative ultrasonography showed that true lumen in the right common carotid artery was compressed by thrombosed false lumen.

Aortic repair for AAAD was planned because the cerebral condition was well-stabilized. Edaravone was administered for the first 2 weeks for extensive cerebral infarction, while antiplatelet or anticoagulant drugs were not used because of the sudden onset of aortic rupture. Strict blood pressure control continued, and the cerebral edema was reduced. She recovered significantly communicate by nodding and speaking some words (GCS E4V3M6). CT revealed a 62-mm dissecting ascending aorta and right common carotid artery with a patent false lumen. Nine weeks after admission, aortic repair of AAAD was performed. Under general anesthesia, cardiopulmonary bypass (CPB) was established with right femoral arterial cannulation and right atrial venous drainage. As a routine procedure, another CPB arterial cannula was inserted into the true lumen of the ascending aorta, which was confirmed by direct echography. The patient was gradually cooled to a tympanic temperature of 22 °C and a bladder temperature of 25 °C. Next, circulatory arrest was induced in the head-down potion while the CPB venous drainage was stopped. With temporary retrograde systemic perfusion through the CPB venous cannula, the ascending aorta was opened. Antegrade selective cerebral perfusion (SCP) of the three arch vessels was started.

**Fig. 3:**
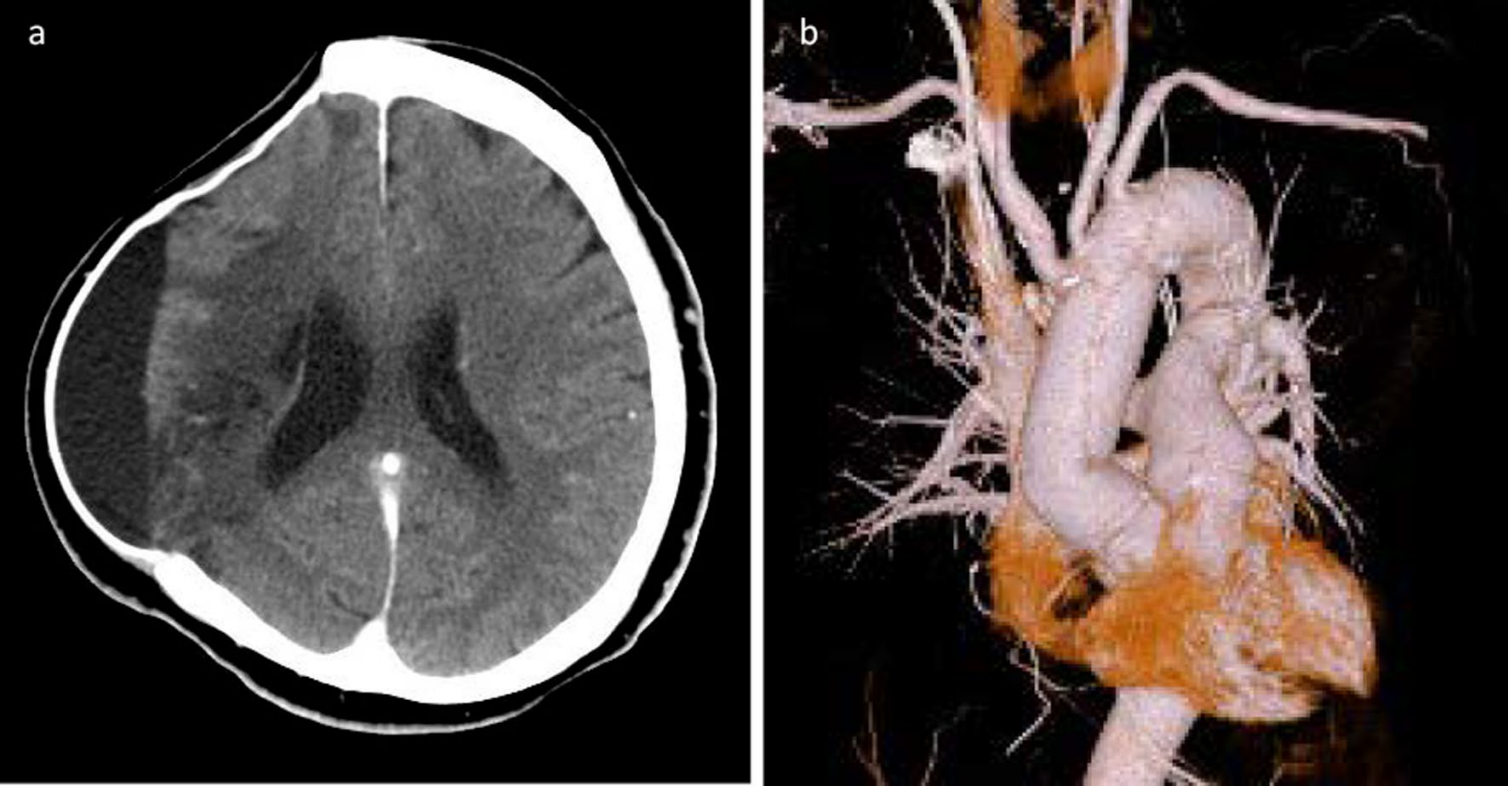
Postoperative CT shows the graft-replaced ascending aorta (**a**) and a moderate amount of cerebral spinal fluid (**b**)

Its target flow rate, its pressure of the bilateral radial arteries and the SCP cannula tip into the left common carotid artery, and its temperature were set at 500–600 ml/min (10–12 ml/kg/min), 40–50 mmHg, and 20 °C, respectively. Near-infrared spectroscopy (NIRS) was used to monitor regional cerebral oxygen saturation (rSO_2_), reflecting perfusion of the bilateral frontal lobe. Primary entry was found in the lesser curvature of the proximal aortic arch. Hemiarch replacement resecting the primary entry was performed using an open distal anastomosis with a Gelweave graft (Terumo-Vascutek Inc, Scotland, UK) of 28 mm. The CPB weaning was uneventful. The duration of SCP, circulatory arrest, CPB, and operation were 28 min, 38 min, 180 min, and 369 min, respectively. No abnormal findings of rSO_2_, including differences between the right and left frontal head, were observed during the open aortic repair or stay in the intensive care unit (ICU). The patient was extubated the day following surgery. Postoperative brain CT showed an increased amount of cerebrospinal fluid in the cranial space, but no new neurological deficits occurred (Fig. 3a). Enhanced CT demonstrated that the dissected ascending aorta was graft-replaced and the true lumen of the brachiocephalic artery and right common carotid artery was patent (Fig. 3b). With the help of intensive rehabilitation, she recovered sufficiently to communicate with some words and pull herself up (MMT 5). Eventually, she went back to the previous hospital to undergo cranioplasty on postoperative day 22. At present, she has recovered further to have daily conversations and come to the hospital using a wheelchair.

## Discussion

The International Registry of Acute Aortic Dissection (IRAD) data revealed that 4.6% of AAAD patients presented with cerebrovascular accidents and 2.9% of those patients presented with coma [1]. Another report also suggested that neurological dysfunction was observed in 20.3% of patients with AAAD [2]. A complicated stroke has a strong impact on morbidity and is associated with higher rates of postoperative complications and a significantly longer hospital stay [3]. Pacini et al. suggested that dissection of supra-aortic branches was an independent predictor of postoperative cerebral morbidities with an estimated odds ratio of 2.18 [4]. Such cerebrovascular events are a severe problem that need to be solved in order to improve the prognosis of patients with AAAD. In this case, the preoperative image examination revealed that the false lumen in the right common carotid artery was thrombosed and the right middle cerebral artery was occluded because of static cerebral malperfusion due to AAAD.

Incidence of neurologic events during CPB has been found in 2.8% of patients [5]. Surgical repair for AAAD requires CPB and full heparinization, frequently with concurrent deep hypothermia and circulatory arrest. This can lead to hemorrhagic conversion, which results in worsening of neurological outcomes. Reperfusion injuries also occur in the cerebral ischemic regions. In the present case, the initial diagnosis was isolated extensive cerebral infarction without a diagnosis of AAAD. It developed into hemorrhagic infarction with brain edema, and emergent decompressive craniectomy was performed without hesitation. After the definitive diagnosis of acute extensive cerebral infarction due to critical cerebral malperfusion of AAAD, there was no choice over strict blood pressure control and brain protection therapy, given the problem of brain hemorrhage and edema caused by emergent aortic repair.

Our concern was that, while being on standby for elective surgery, emergent or urgent surgery might be required in the setting of rupture or progression of dissection, which would result in more severe cerebral dysfunction. Strict blood pressure control is necessary to prevent rupture and progression of the dissection. In the present case, we attempted to reduce the possibility of lethal aortic events by using strict therapies for hypertension and brain edema for 9 weeks. Since the diameter of the ascending aorta was 62 mm and sufficient recovery of her neurological conditions had been recognized, elective aortic repair was finally decided upon.

Patients with severe brain injury and edema usually require emergent surgical intervention; decompressive craniectomy is the procedure of choice to release intracranial pressure and open the brain cavity for further surgical treatment [6]. There is growing evidence supporting the efficacy of decompressive craniectomy, including the reduction of intracranial pressure and prevention of brain edema [7]. The defective dura area may be left open or sealed with artificial dura substitutes. In addition, during prolonged CPB with deep hypothermia, brain edema theoretically worsens [8]. As far as we investigated, regarding brain herniation during and after aortic repairs, several case reports described that decompressive craniectomy is required after aortic repair for AAAD [9–11], whereas there are few reports of aortic repair after decompressive craniectomy for critical cerebral infarction secondary to AAAD. There is a case report of delayed repair of AAAD 4 months after closing craniotomy [12]. Aortic repair with craniectomy left open, as in the present case, is extremely rare. Regarding brain protection for critical cerebral malperfusion due to AAAD, prompt ligation of the right common carotid artery to prevent brain edema and bleeding during open aortic repair was recommended as a useful option [13]. In contrast, Okita et al. recommended direct right common carotid artery cannulation for patients with cerebral malperfusion [14]. Definitive treatment for AAAD patients with severe cerebral malperfusion has not yet been established.

In the aortic repair, the core temperature was set to a bladder temperature below 25 °C (lower than the usual 28 °C) for more meticulous brain protection. In addition, the SCP perfusion temperature was also lower (20 °C) than the usual temperature of 23 °C. For such a high-risk case, limited hemiarch replacement was performed to reduce the cerebral ischemic time because the primary entry was located at the lesser curve of the proximal arch. Fortunately, the patient did not have a new neurological disorder, although an increased amount of cerebrospinal fluid in the cranial space was recognized on the postoperative CT scan. Owing to CPB and SCP with deep hypothermia, preoperative open decompression craniectomy with a compliant artificial dura seemed to be effective in releasing the intracranial pressure. Moreover, continuous monitoring of bilateral rSO_2_ by NIRS was also useful for real-time monitoring of cerebral perfusion during the ICU stay as well as the stay in the operating room.

## Conclusions

We report a rare aortic repair following initial decompressive craniectomy for extensive hemorrhagic cerebral infarction with optimal conservative therapy for AAAD. This treatment strategy might be a useful option in the setting of critical cerebral malperfusion due to AAAD having no emergency surgical indication due to some delay about its diagnosis and surgical referral.

## Data Availability

No additional data.
